# Impact of E-Liquids on Phagocytosis, Escape and Invasion of *Porphyromonas gingivalis*

**DOI:** 10.3390/pathogens15080870

**Published:** 2026-08-20

**Authors:** Maya Tabakha, Raivat Shah, Sophie Tomov, Juliette R. Amram, Dominic Palazzolo, Giancarlo A. Cuadra

**Affiliations:** 1 Department of Biology, Muhlenberg College, 2400 W. Chew Street, Allentown, PA 18104, USA; mayatabakha@gmail.com (M.T.); stomov@muhlenberg.edu (S.T.); jamram@muhlenberg.edu (J.R.A.); 2 Medical School, University of Michigan, 1500 E. Medical Center Drive, Ann Arbor, MI 48109, USA; raivat@med.umich.edu; 3 DeBusk College of Osteopathic Medicine, Lincoln Memorial University, Harrogate, TN 37752, USA; domenico.palazzolo@lmunet.edu

**Keywords:** M1 macrophages, pseudopodia, phagocytosis, microbial escape, microbial invasion, *P. gingivalis*, *E. coli*, oral epithelial cells, periodontitis, flavored e-liquids

## Abstract

This study seeks to understand the effects of electronic cigarettes on the oral microenvironment. Macrophages are paramount in the oral innate immune response against pathogens. *Porphyromonas gingivalis* is a Gram-negative bacterium that invades oral epithelial cells, escapes phagocytosis and is associated with the progression of periodontitis. We hypothesize that e-liquid treatments enhance *P. gingivalis*’s escape from post-macrophage phagocytosis and subsequent invasion into oral epithelial cells. THP-1-derived macrophages were polarized to M1 ± 1% e-liquids (±menthol or cinnamon). The M1 cellular morphology and surface area were determined after e-liquid treatments. *Escherichia coli* or *P. gingivalis* were added to M1 macrophages ± e-liquids. Phagocytosis and escape were determined by multiple assays. After e-liquid treatments and phagocytosis, OKF6/TERT-2 cells (oral epithelial cells) were exposed to M1 macrophages with intracellular *P. gingivalis* to evaluate escape and invasion. The results show that cinnamon e-liquids alter M1 morphology by diminishing pseudopodia extensions and actin stress fibers, which persist after a second polarization attempt. Phagocytosis of *P. gingivalis* by e-liquid-treated macrophages also decreases. Furthermore, *P. gingivalis* absolute escape and OKF6/TERT-2 cell invasion increase after menthol and cinnamon e-liquid treatments, respectively. Thus, following e-liquid exposure, phagocytosis and retention of *P. gingivalis* deteriorate.

## 1. Introduction

Despite over 20 years of being marketed as a safer alternative to smoking, the use of electronic cigarettes (e-cigs) may not be as safe as once believed [1,2,3]. Newer e-cig devices and e-liquid flavor formulations continue to attract users, especially adolescents [4,5,6], creating a public health concern. Recently, e-cig use, commonly known as vaping, has exponentially increased, especially among adolescents. As of 2024, 7.8% (1.21 million) of high school students and 3.5% (410,000) of middle school students reported the use of e-cigs [1].

In its simplest design, an e-cig consists of a battery, which supplies the power to a heating coil within a reservoir in which the e-liquid can be aerosolized. The e-liquid components are typically a varying mixture of propylene glycol (PG), vegetable glycerol (VG), nicotine (freebase or nicotine salt) and a myriad of flavoring agents [7,8]. While e-liquids contain relatively fewer toxic chemicals than combustible tobacco [9], this does not mean that e-liquids are innocuous. First, since e-liquids contain nicotine, a highly addictive substance, and enticing flavors, users become addicted to vaping, leading to long-term use [10]. Furthermore, nicotine is known to interact with nicotinic acetylcholine receptors in the central nervous system, playing a vital role in brain development and leading to changes in learning and motivation, as well as addiction [11]. Second, heating and aerosolization of e-liquids can lead to the formation of tobacco-specific nitrosamines (TSNAs) and multiple volatile organic compounds (VOCs) such as aldehydes and carbonyls [9,12,13]. Third, the high temperatures of the heating coils can lead to leaching of heavy metals such as nickel, lead and chromium when using e-liquid-generated aerosols [14,15,16,17].

Physiological and biochemical studies reveal that vaping is associated with various forms of toxicity. For example, when exposed to e-cig-generated aerosols, the cell viability and alveolar blood barrier integrity of airway epithelial cells are decreased and inflammatory cytokine release is altered [18,19]. In addition, airway macrophage phagocytosis is also decreased upon e-liquid exposure [20,21,22]. Furthermore, the mucociliary function of the airway is compromised upon vaping [23,24,25]. While the effects on the airway under vaping exposure are extensive, less research has been done concerning the oral microenvironment, which is perplexing given that the oral cavity is the initial site of exposure upon vaping. In the present study, we start by examining the effects of e-liquids on macrophage phagocytosis using an oral pathogen.

The oral cavity houses over 700 species, meaning it has the second largest microbiome after that of the gut [26,27]. Its composition is highly dynamic, undergoing constant shifts based on diet, oral health habits, microbial interactions and pH fluctuations [28]. Dental plaque is one of the most common biofilms occurring in the oral cavity [29]. Microbial colonization begins with oral commensal bacteria maintaining microbial homeostasis but their accumulation and maturation could lead to the attachment of secondary colonizers and pathogenic bacteria [30,31,32].

One of these bacteria is the oral pathogen *Porphyromonas gingivalis*, a Gram-negative anaerobe that plays a significant role in oral health, particularly in the development of chronic periodontitis and other systemic diseases such as atherosclerosis, Alzheimer’s disease, rheumatoid arthritis, and diabetes [33,34]. Periodontitis is an inflammatory condition of the gingiva and is characterized by the deterioration of surrounding structures supporting the tooth, including the alveolar bone and periodontal ligament [35,36]. *P. gingivalis* possesses several virulence factors such as lipopolysaccharides (LPSs), fimbriae, and gingipains, all of which contribute to its pathogenicity and the destruction of the gum tissue [35,36]. *P. gingivalis* can also disrupt the host immune response by producing gingipains that degrade immune components such as cytokines and immunoglobulins and using fimbriae to invade epithelial and endothelial cells [37]. Uniquely, this oral pathogen is also able to avoid the immune response by escaping macrophage phagocytosis [38].

Pro-inflammatory macrophages, such as those in the oral cavity, are ubiquitous and play a key role in the immune response, particularly in the phagocytosis of microbes and other foreign materials [39]. These bone marrow-derived and highly plastic myeloid cells circulate in the blood as monocytes and extravasate into peripheral tissues where inflammation is taking place [40]. At this point, monocytes undergo a polarization pathway in response to external cues, becoming naïve M0 macrophages [41,42]. These polarization states are classified as pro-inflammatory and anti-inflammatory or M1 and M2 macrophages respectively, both derived from the naïve M0 macrophage [43]. Polarization enables these cells to carry out their defense roles such as supporting tissue repair and growth (M2) or carrying out phagocytosis and bacterial defense (M1) depending on the environmental stimuli, which makes these cells highly adaptable.

In vitro, M1 macrophage activation is induced through bacterial cell wall component LPSs and pro-inflammatory cytokines like tumor necrosis factor (TNF)-α or interferon IFN-γ [44]. M1 macrophage phagocytosis occurs through a multi-step mechanism which begins with the recognition of microbes tagged with immunoglobulin G (IgG) by Fc*γ* receptors, triggering an intracellular signaling cascade [45]. This eventually leads to the extension of pseudopodia—a major characteristic of M1 morphology and function [46,47]. Specifically, this process is mediated by IgG antibodies recognized by Fcγ receptors. This activates Src kinases which phosphorylate Immunoreceptor Tyrosine-based Activation Motifs which then trigger further signaling pathways [40]. Ultimately, this promotes actin polymerization and cytoskeleton rearrangement leading to the extension of pseudopodia and phagosome formation within the macrophage [48].

In the oral environment, M1 macrophages, located in the lamina propria below the epithelium, are among the first to interact with microbes, and this interaction is key in the development and progression of periodontal disease. For example, in a mouse model of *P. gingivalis*-mediated periodontal disease, reducing macrophage activity resulted in a decrease in bone loss [49]. Therefore, M1 macrophages are a reasonable model to study the innate immune response, given the M1 involvement in host–microbe interactions and phagocytosis. In order to study human macrophage immunobiology, the THP-1 cell model has been extensively employed [50]. In a previous study from our group, THP-1-derived human M0 macrophages were polarized to the M1 subtype in the presence of 1% e-liquids and exhibited an atypical phenotype, as well as alterations in gene expression and cytokine production, particularly when exposed to cinnamon- or menthol-flavored e-liquids [51]. Given these effects, it is imperative to investigate whether or not morphological changes in M1 macrophages are reversible with a second polarization attempt and if key functions (phagocytosis) are compromised. A Gram-negative aerobe, *Escherichia coli*, serves as a model organism for preliminary assessments of macrophage–bacteria interactions due to this microbe’s ability to grow rapidly and the ease with which its genome can be manipulated, specifically through transformation with green fluorescent protein (GFP) plasmids [52].

Given the invasive ability of *P. gingivalis* and the effects of e-liquids on macrophage differentiation and immune signaling, it is imperative to explore macrophage–bacteria interactions while exposed to e-liquids. Therefore, the aim of this study is to determine how e-liquids affect macrophage phagocytosis of *E. coli* as a preliminary proof-of-concept, and to explore *P. gingivalis* phagocytosis and escape from M1 macrophages, followed by its invasion into oral epithelial cells when exposed to e-liquids. We hypothesize that when exposed to e-liquids, macrophage phagocytosis will be diminished and *P. gingivalis* escape and invasion will be enhanced. If *P. gingivalis* can escape phagocytosis in healthy individuals, vaping may enhance this process, leading to greater invasion into oral epithelial tissues and higher risk of infection. This research addresses the long-term health risks of vaping and provides a potential link between vaping and periodontal disease.

## 2. Materials and Methods

### 2.1. E-Liquid Preparation

E-liquids consist of 50% PG, 50% VG, 20 mg/mL of (S)-(−)-nicotine, and 99% (Alpha Aesar, Tewksbury, MA, USA) and 5% *v*/*v* cinnamon (Liquid Nicotine Wholesalers, Phoenix, AZ, USA) or menthol (Vapor Vapes, Sand City, CA, USA). Flavorless e-liquids were prepared similarly, omitting the flavors. All e-liquids were stored at 4 °C until required.

### 2.2. THP-1 and OKF6/TERT-2 Culture Methods

THP-1 cells, kindly shared by Dr. Angela Brown from Lehigh University (Bethlehem, PA, USA), were routinely cultured in Roswell Park Memorial Institute (RPMI) medium containing 10% Fetal Bovine Serum and 1:100 penicillin/streptomycin at 37 °C, with 5% CO_2_. THP-1 cell maintenance, including passaging, trypan blue live/dead staining, cell counting and dilutions, was performed in a biosafety flow hood. THP-1 cell density was maintained between 0.2 and 1.2 × 10^6^ cells/mL.

**Figure 1:**
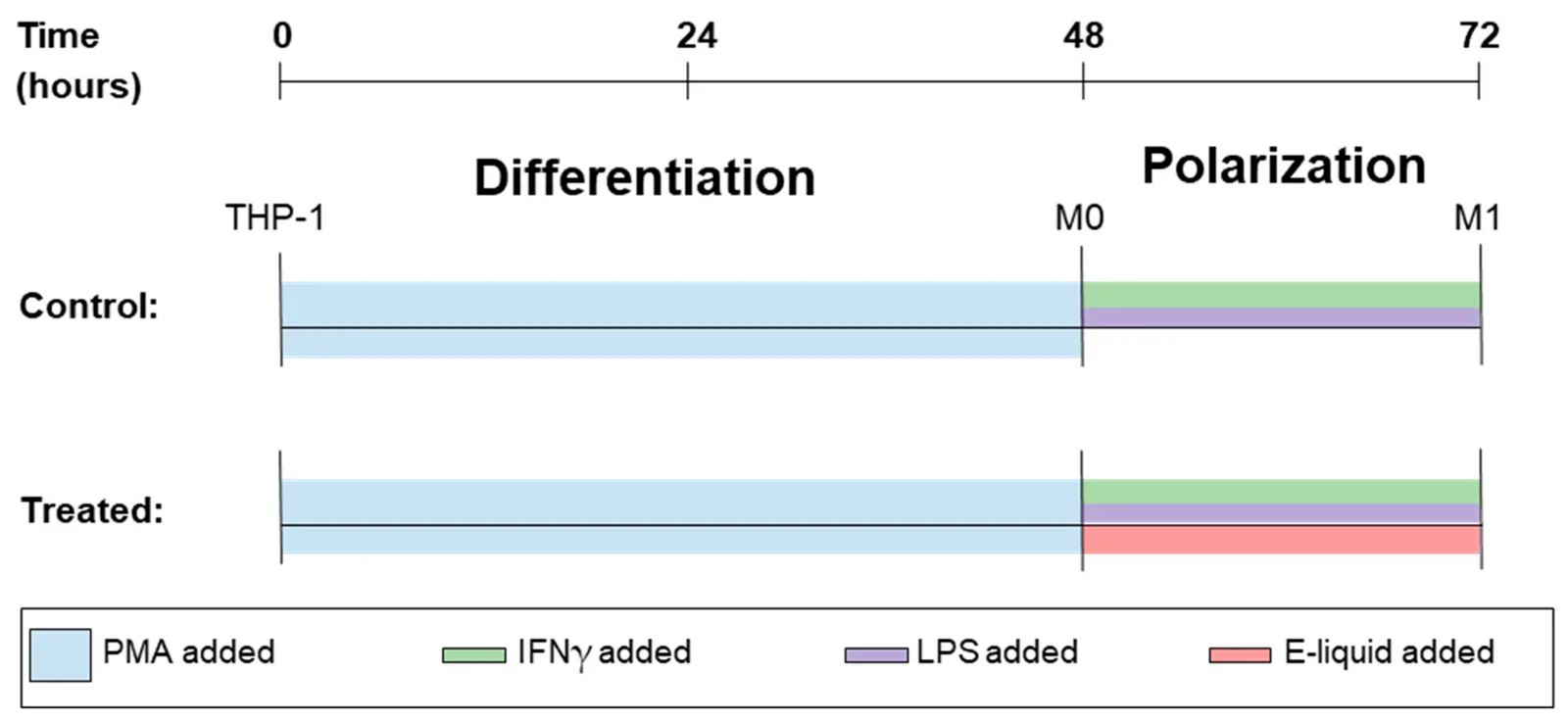
Progression of THP-1 precursor cells to M0 and to M1 macrophages with the addition of PMA, IFNγ, and LPS, treated with e-liquids ± flavors.

For experiments, THP-1 cells were cultured and differentiated following previously established protocols [51]. Briefly, THP-1 cells were seeded into 24-well plates at a density of 0.2 × 10^6^ cells/mL with a final volume of 1 mL RPMI containing 200 nM of phorbol 12-myristate 13-acetate (PMA) and differentiated for 48 h to M0 macrophages. Following differentiation, media with PMA were removed and M0 macrophages were polarized to M1 by adding 1 mL of RPMI containing 100 ng/mL of LPSs and 20 ng/mL of IFNγ per well. Simultaneously, 1% (*v*/*v*) flavorless or menthol- or cinnamon-flavored e-liquid was added during M1 polarization, following our published protocol [51]. The final percentages of e-liquid components in the media were 0.5% PG, 0.5% VG, 0.2 mg/mL nicotine and 0.05% flavoring agent. Cells were then incubated for an additional 24 h under the same conditions (Figure 1).

OKF6/TERT-2 cells were routinely cultured in Keratinocyte Serum-Free Medium (KSFM) at 37 °C and 5% CO_2_ and passaged at least once a week. For invasion experiments (Section 2.9), cells were seeded at 0.1 × 10^6^ cells/mL in 24-well plates and incubated for 24 h under the same conditions. After 24 h the KSFM was replaced with DFK prepared by mixing DMEM/F12 and KSFM at 1:1 (*v*/*v*), as previously described [53]. OKF6/TERT-2 cells were cultured for three to five days in DFK until confluent.

### 2.3. Quantification of M1 Surface Area with ImageJ

M1 macrophages were polarized in the presence of 1% e-liquids ± flavors as above. Representative light microscopy images of each condition were taken at 100× magnification. Quantification of cellular surface area, in pixels, was accomplished using ImageJ (v. 1.54g) by outlining individual cells.

### 2.4. Second-Attempt Polarization of M1 Macrophages Following Cinnamon Flavoring

Based on our previous results, M1-polarized macrophages treated with 1% e-liquids with five different flavors remained viable and the morphology in the presence of cinnamon e-liquids did not present pseudopodia [51]. To determine if this morphological defect induced by cinnamon e-liquids is reversible, macrophages were first polarized to the M1 subtype in the presence of cinnamon-flavored e-liquids, as described above (Section 2.2, Figure 1). After 24 h of 1% cinnamon-flavored e-liquid treatment, LPSs and IFNγ, M1 macrophages were imaged at 100× magnification. One group of cells exposed to cinnamon were then washed twice with 1 mL PBS to remove the cinnamon e-liquid. Then, media with LPSs and IFNγ were added to the cells in a second attempt to polarize to the M1 subtype. Cells were imaged at 100× magnification for up to seven days to observe changes in morphology. M1 control cells and cinnamon-exposed cells were cultured alongside as positive and negative controls, respectively, for the M1 phenotypic morphology.

### 2.5. Bacterial Culturing Methods

The two bacterial strains in this study are *E. coli* DH5α GFP+ (Beckton, Dickinson Company, Franklin Lakes, NJ, USA) and *P. gingivalis* W83 (from Dr. Progulske-Fox of University of Florida College of Dentistry). *P. gingivalis* is grown at 37 °C anaerobically in a BACTRON Anaerobic Chamber (Sheldon Manufacturing, Inc., Cornelius, OR, USA) on tryptic soy agar (TSA) containing 5% sheep’s blood and 1 μg/mL menadione (vitamin K) and selected with 30 μg/mL gentamicin or tryptic soy broth with 5 μg/mL porcine hemin and 1 mg/mL yeast extract (TSBY) containing the same concentrations of menadione and gentamicin as detailed above. *E. coli* was grown at 37 °C with 5% CO_2_ in TSA and TSBY containing 1 mg/mL arabinose for GFP expression and 1 μg/mL menadione and selected with 100 μg/mL ampicillin.

### 2.6. Phagocytosis Assay

Phagocytosis was performed following our previously published protocols [38] with a few modifications. Briefly, *E. coli* or *P. gingivalis* were grown in TSBY overnight to a stationary phase and adjusted to OD = 1.0 (10^9^ bacteria/mL). Then, bacteria were washed twice in PBS and diluted in antibiotic-free RPMI to a final concentration of 20 × 10^6^ bacteria per mL. Prior to the phagocytosis assay, M0 macrophages were polarized to the M1 subtype in the presence of 1% e-liquids ± flavors (Figure 1). Twenty-four hours post-treatment, cells were washed twice with PBS and incubated with 1 mL of bacterial suspension (MOI = 100) for 1 h at 37 °C, with 5% CO_2_. Following the 1 h incubation, excess bacteria were removed by washing macrophages twice with 1 mL PBS. To kill any extracellular bacteria, RPMI spiked with 300 μg/mL gentamicin, 200 μg/mL metronidazole and 5× penicillin/streptomycin, referred to as killing medium, was added to the cultures for 1 h under the same conditions. Then, cells were washed twice to remove excess antibiotics and lysed with 1 mL sterile water to release intracellular bacteria. Lysates containing bacteria were then serially diluted, plated on their respective agars and grown as indicated above (Section 2.5). Colony-forming units (CFUs) were quantified by colony counting.

### 2.7. qPCR

Phagocytosis assays were performed as described above (Section 2.6). After the killing media step, cells were washed twice with PBS and DNA was isolated using the DNeasy Blood & Tissue Kit (QIAGEN, Hilden, Germany. cat: 69506), following the manufacturer’s instructions. Next, the NanoDrop 2000c (Thermo Scientific, Waltham, MA, USA) was used to determine DNA concentrations. To quantify all macrophages and bacteria per well, Ct values were determined through a real-time simultaneous amplification of the 18S and the 16S rRNA, respectively, in the same wells, using VIC-labeled 18S and FAM-labeled 16S TaqMan assays and the StepOnePlus Real-Time PCR machine (Applied Biosystems, Foster City, CA, USA). The relative abundances of 16S and 18S DNA were calculated using the 2^−ΔCt^ analysis.

### 2.8. Fluorescent Microscopy

To visualize actin filaments and stress fibers required for pseudopodia formation, THP-1 cells were cultured on coverslips, differentiated and polarized to M1 macrophages under e-liquid treatments as indicated above (Figure 1). Macrophages were fixed with 4% formaldehyde at room temperature for 20 min. Cells were then permeabilized with 0.1% Triton X-100 in PBS and stained with phalloidin–rhodamine to visualize actin filaments in red. Fluoromount-G™ Mounting Medium with DAPI was used to mount the cells on glass slides and stain the nuclei blue. The Nikon Eclipse TE2000-U microscope (Nikon, Melville, NY, USA) was used to image the cells at 200× with an ultraviolet laser for DAPI (nuclei) emission and a green laser for rhodamine (actin) emission. Nuclei and actin images from the same field of view were then merged using ImageJ (v. 1.54g). Additionally, phagocytosis experiments with GFP+ *E. coli* as indicated above (Section 2.6) were also performed on coverslips. After cells were exposed to killing media, they were washed and processed for fluorescent microscopy as indicated above. Cells and bacteria were visualized at 1000× magnification using a blue laser for GFP (*E. coli*) emission, and the ultraviolet and green lasers for nuclei and actin filaments, respectively. Again, ImageJ was used to merge nuclei, *E. coli* and actin images from the same field of view.

### 2.9. P. gingivalis Escape from M1 Macrophages and Invasion into OKF6 Cells

THP-1 cells were cultured, differentiated to M0 and polarized to M1 macrophages with and without 1% e-liquids ± flavors as above (Figure 1). Phagocytosis assays with *P. gingivalis* were performed as indicated above (Section 2.6). After cells were exposed to killing media, coverslips with macrophages containing intracellular *P. gingivalis* were washed twice with PBS and cells were cultured in antibiotic-free RPMI for 1 h to allow any bacteria to escape into the supernatant. After the hour, supernatants were collected, serially diluted and plated on blood agar. *P. gingivalis* CFUs were grown as indicated above (Section 2.5) and quantified by CFU counting.

For escape and invasion assays, OKF6/TERT-2 cells were grown with KSFM and DFK in 24-well plates to confluency (Section 2.2). THP-1 cells were cultured in coverslips, differentiated to M0 and polarized to M1 macrophages with and without e-liquids ± flavors as above (Section 2.2). After the phagocytosis assay, coverslips with macrophages and intracellular *P. gingivalis* were lifted with fine sterile forceps and placed on top of the OKF6/TERT-2 cells, allowing both cell types to face each other in antibiotic-free DFK. This allowed *P. gingivalis* to escape and potentially invade OKF6/TERT-2 cells. After 1 h of incubation, coverslips were removed and OKF6/TERT-2 cells were washed twice with PBS. DFK killing media (containing the same antibiotic as above) were then added to the OKF6/TERT-2 cells to kill extracellular bacteria for 1 h. Cells were washed twice, removing excess antibiotics, and lysed with sterile water. Lysates were serially diluted and plated on blood agar to obtain CFU counts.

### 2.10. Statistical Analyses

For each treatment group in all experiments, the means ± standard error of the means (SEM) were calculated. Statistical significance between treatment groups for all experiments was determined using One- and Two-Way ANOVA followed by Bonferroni post hoc analysis. Differences were considered statistically significant when *p* < 0.05. Experiments were all repeated at least four times with three biological replicates each. For each replicate, CFU counts were plated in triplicates.

## 3. Results

### 3.1. Visualization of Actin Filaments and Quantification of M1 Surface Area

**Figure 2:**
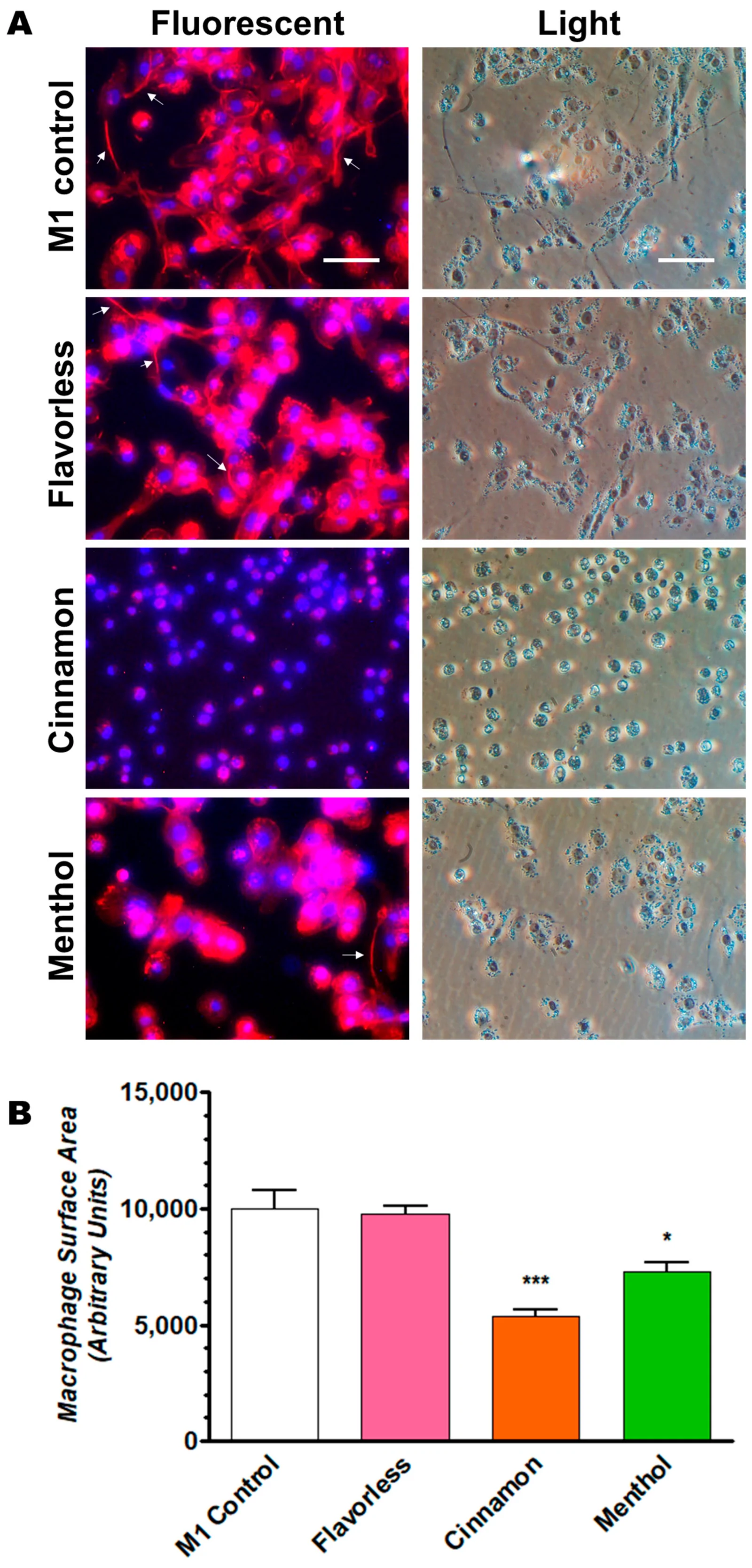
(**A**) Fluorescent and light micrographs of THP-1-derived M1 macrophages polarized with and without 1% e-liquids ± flavors. Actin was stained with phalloidin red and nuclei were stained with DAPI blue. Stress fibers are indicated by white arrows. Representative micrographs from four independent experiments are presented at 200× magnification. White bars represent 100 µm. (**B**) Quantification of cellular surface area in pixels (ImageJ) of M1 macrophages polarized with e-liquids ± flavors. Each bar represents the mean ± SEM where n = 10 to 15 cells per image from four independent experiments. Significance determined by One-Way ANOVA. * = *p* < 0.05 and *** = *p* < 0.001.

Evaluations of cellular morphology and actin filament activity in polarized macrophages treated with e-liquids ± flavors are shown in Figure 2A. As expected, M1 control and flavorless e-liquid-treated cells reveal multiple pseudopodia, which is consistent our previous results [51], and pseudopodia are co-existent with stress fibers (Figure 2A). In contrast, menthol- and cinnamon-flavored e-liquid-treated M1 macrophages present few to no stress fibers, respectively. In Figure 2B, cell surface areas (in pixels) are determined after M1 polarization with and without e-liquids and are consistent with the microscopic images shown in Figure 2A. Macrophages treated with menthol- or cinnamon-flavored e-liquids exhibit significant decreases in surface area (*p* < 0.05), concomitant with reduced pseudopodia extensions compared to M1 controls (Figure 2). These results confirm that cinnamon- and menthol-flavored e-liquids significantly alter M1 macrophage morphology.

### 3.2. Second Polarization Attempt of M1 Macrophages After Treatment with Cinnamon-Flavored E-Liquid

**Figure 3:**
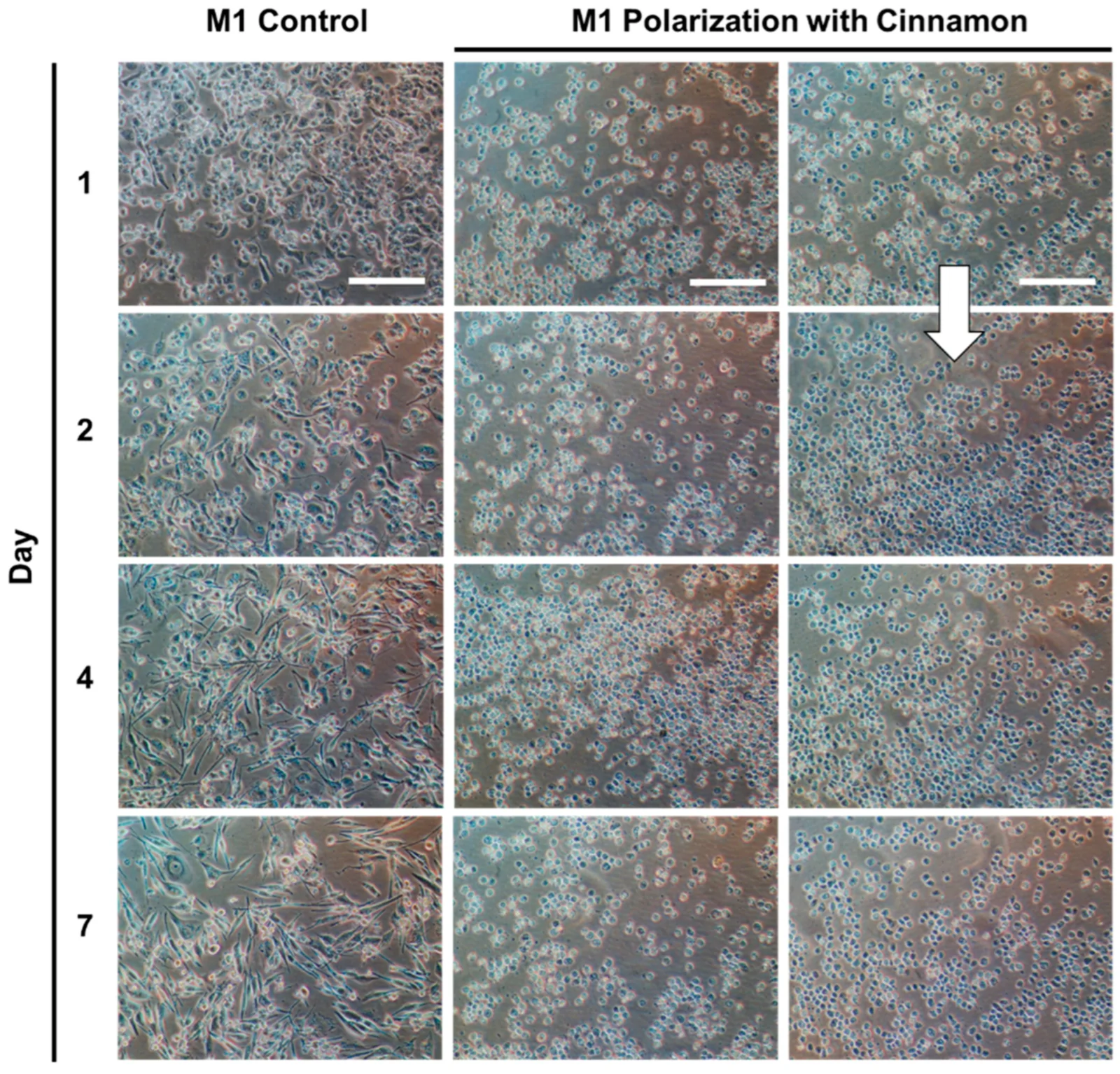
Micrographs of M1 macrophages polarized with and without cinnamon-flavored e-liquid. White arrow indicates that cinnamon was washed away and cells were re-exposed to LPSs and INF-γ by day 2, attempting to rescue the M1 phenotype. Micrographs are representative images from four independent experiments. White bars indicate 100 µm.

In order to assess the reversibility of the most pronounced morphological alterations in M1 macrophages induced by e-liquid exposure, cells polarized in the presence of cinnamon e-liquids were washed and re-exposed to LPSs and INF-γ in an attempt to recover the M1 phenotype. On day 1 of the experiment, M1 cells polarized in the presence of cinnamon e-liquids exhibited a rounded morphology lacking the pseudopod extensions characteristic of typical M1 cells (Figure 3) [51]. While control cells continue to extend prominent pseudopodia over seven days, cinnamon treatment renders cells with a rounded morphology (Figure 3). This morphology persists even after washing the cinnamon e-liquid out of the cultures and re-exposing the cells to LPSs and INF-γ over six days (Figure 3, white arrow). This indicates that the morphological alterations caused by cinnamon e-liquids persist over the duration of this experiment.

### 3.3. Macrophage and E. coli Interactions Quantified by qPCR, CFU and Fluorescent Microscopy

In order to determine whether phagocytosis of *E. coli* was hindered by e-liquid treatments, the levels of *E. coli* associated with M1 macrophages were evaluated with e-liquid treatments via qPCR, CFU counting, and fluorescent microscopy. Table 1 shows quantitative results following *E. coli*–macrophage interactions. Ct value results in Table 1 show that the macrophage DNA quantification is similar across all conditions, indicating that the total number of macrophages per well stays consistent throughout the assays. To display the change in phagocytic efficacy between treatment groups, the ratio of bacteria:macrophages is also displayed in Table 1 (also see Table S1), where the M1 control is 3.74 ± 0.41 bacteria per macrophage. By comparison, the ratios of bacteria:macrophages in flavorless and cinnamon e-liquid treatments are significantly less (*p* < 0.05). Menthol treatment, however, induces a lower but not significant ratio of bacteria to macrophages compared to the M1 control (Table 1 and Table S1). Since all cultures begin with 200,000 macrophages per well and there are 3.74 times more bacteria in the M1 control, there is an estimated total of 748,900 ± 81,950 *E. coli* bacteria associated with these macrophages. Although these values are not validated by a standard curve, they are still useful to compare ratios of bacteria and macrophages. Based on these calculations, there are significantly (*p* < 0.05) less total estimated bacteria associated with the macrophages exposed to flavorless and cinnamon-flavored e-liquids, but not menthol, demonstrating decreased binding to bacteria under these treatments.

After macrophage lysing and CFU counting, intracellular *E. coli* was quantified post-phagocytosis (Table 1). On average, M1 control macrophages engulfed 19,833 ± 1148 *E. coli* bacteria/well. Macrophages polarized with e-liquids had significantly (*p* < 0.001) less intracellular bacteria compared to the M1 control. Of the estimated total bacteria associated with macrophages, less than 3% were engulfed by macrophages. Furthermore, macrophages treated with e-liquids including flavors engulfed significantly (*p* < 0.001) less bacteria as a percentage of the estimated total compared to the M1 control and flavorless treatment. These results suggest that e-liquid treatments, especially those with cinnamon and menthol flavors, alter the macrophage–*E. coli* interactions, decreasing the engulfment of bacteria.

**Figure 4:**
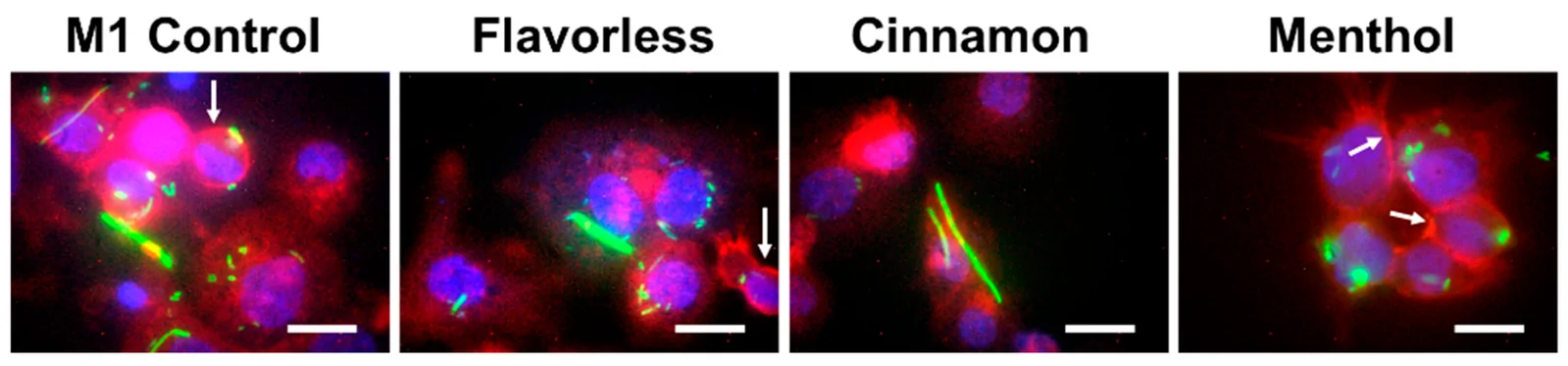
Micrographs of THP-1-derived M1 macrophages polarized with and without 1% e-liquids ± flavors. Actin is stained with phalloidin red, nuclei are stained with DAPI blue and GFP+ *E. coli* is green. Stress fibers are indicated by white arrows. Representative micrographs from four independent experiments were taken at 1000× magnification.

Following quantitative assessment of *E. coli* and macrophage interactions after M1 polarization, the amount of GFP+ *E. coli* interacting with M1 cells was assessed by fluorescent microscopy, as depicted in Figure 4. All three e-liquid-treated groups, especially cinnamon, present less *E. coli* (green) than the M1 control, supporting the findings in Table 1. In addition, actin-derived stress fibers (red) are visible as polymerized filaments around the nuclei (blue) in all conditions except for the cinnamon treatment, which shows actin but no stress fibers.

### 3.4. Quantification of Intracellular, Escape and Invading P. gingivalis After Phagocytosis Assays

**Figure 5:**
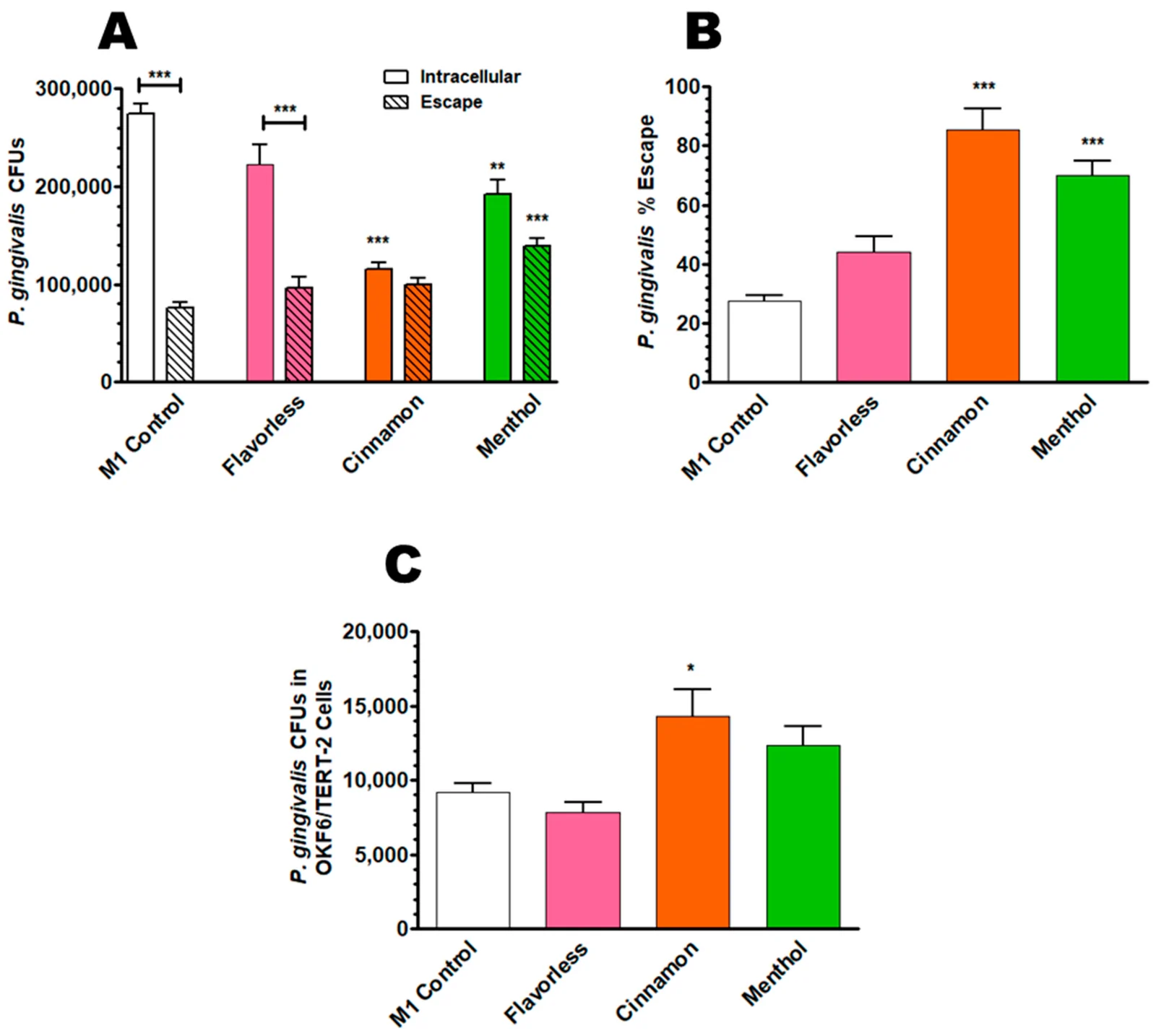
(**A**) *P. gingivalis* intracellular and escaping CFU counts after a phagocytosis and escape assay. (**B**) The escaping CFU counts presented as percentages of the respective intracellular counts. (**C**) CFU counts of *P. gingivalis* invasion into OKF6 cells following macrophage phagocytosis escape. Bars and error bars represent average and SEM, respectively, with n = 6 to 11. Significance determined by One- and Two-Way ANOVA. * = *p* < 0.05, ** = *p* < 0.01, and *** = *p* < 0.001.

To evaluate engulfment and escape of *P. gingivalis*, both intracellular and escaping bacteria were quantified following phagocytosis of e-liquid-treated M1 macrophages through CFU counting (Figure 5A, Table 2 and Table S2). Similar to previous results (18), M1 macrophages are able to engulf *P. gingivalis* at 274,394 ± 10,288 CFU/well. Intracellular *P. gingivalis* after e-liquid treatments results in lower phagocytosis rates; specifically cinnamon- and menthol-flavored e-liquid treatments result in significantly (*p* < 0.01) lower *P. gingivalis* intracellular CFUs (Figure 5A, Table 2 and Table S2). The escape of *P. gingivalis* from M1 macrophages after treatments is also measured by CFU counting. From the M1 control, 75,680 ± 5226 CFUs are able to escape macrophages, echoing previous results [38]. While e-liquid treatments yield slightly higher absolute values of escaping bacteria, only the menthol-flavored e-liquid treatment shows significance (*p* < 0.001) compared to the M1 control. Furthermore, the M1 control and flavorless e-liquid treatment show significantly lower escape levels in comparison to total intracellular CFUs (Figure 5A; *p* < 0.001). On the other hand, no such difference is detected with cinnamon and menthol treatments. Moreover, the escape rate was calculated as a percentage of the total intracellular bacteria with a roughly 28%, 44%, 85% and 70% escape observed from the M1 control, flavorless, cinnamon treatment and menthol treatment, respectively (Figure 5B and Table 2 and Table S2), where the escape percentages for the latter two treatments were significantly (*p* < 0.001) higher compared to the M1 control. Following *P. gingivalis* escape from macrophages, its invasion into OKF6/TERT-2 cells was quantified by CFU counting (Figure 5C, Table 2). *P. gingivalis* bacteria escaping from M1 control macrophages invaded OKF6/TERT-2 at a rate of 9183 ± 1341 CFUs per well. Only after cinnamon e-liquid treatment were *P. gingivalis* CFUs invading OKF6/TERT-2 cells significantly (*p* < 0.05) greater than the M1 control. The results indicate that cinnamon-flavored e-liquid treatment decreases *P. gingivalis* phagocytosis and allows for its escape from macrophages that consequently leads to increased invasion into OKF6/TERT-2 cells.

## 4. Discussion

Phagocytosis is a vital mechanism carried out by macrophages as part of the innate immune response. In the present study, M1 macrophages continue to elongate their pseudopodia several days post-polarization, which is consistent with work by Liang et al. (2022) [54]. Our group has previously shown an alteration in macrophage phenotype during M0 to M1 polarization upon 1% e-liquid exposure [51]. Exposure to cinnamon- and menthol-flavored e-liquids results in altered M1 macrophage morphology (Figure 2A), which agrees with the findings of our previous publication [51]. Cellular actin filaments normally form stress fibers and are visible in M1 control and flavorless-treated macrophages. In contrast, these stress fibers are less obvious in the menthol-flavored e-liquid treatment and are absent in cinnamon-treated macrophages. In addition, menthol-treated cells display shortened pseudopodia extensions, while the cells exposed to cinnamon e-liquids lack pseudopodia altogether. A related study found that neutrophils exposed to flavored e-liquids or their aerosols delivered in the cell culture media present altered morphology and decreased formation of neutrophil extracellular traps [55].

The observed reduction in cellular surface area in this study directly corresponds to the rounded morphology, lack of stress fibers and subsequent absence of pseudopodia extensions after cinnamon and menthol e-liquid treatments (Figure 2). These findings both bolster and explain previous observations from our lab [51], confirming not only that the cellular morphologies are significantly altered, but that the alterations are due to changes in the cytoskeletal structure of the M1 cell (Figure 2).

In light of the morphological alterations in M1 macrophages caused by cinnamon e-liquids (Figure 2), an experiment re-exposing macrophages to LPSs and IFN*γ* after washing away the cinnamon e-liquid was conducted and it was determined that the initial effect of the cinnamon-flavored e-liquid was not reversible within the duration of the experiment. While the M1 control cells continued to extend their pseudopodia over the course of the experiment, as expected [56,57,58], the cells exposed to cinnamon e-liquids during M1 polarization and a subsequent second attempt at M1 polarization in the absence of the e-liquid continued to display a rounded morphology (Figure 3). The persistence of this rounded phenotype, despite the removal of the cinnamon e-liquid and the re-addition of LPSs and IFN*γ*, indicates that the morphological aberrations last at least seven days. These results suggest that cinnamon-flavored e-liquids may cause severe alterations in unknown mechanisms to the cells, begging further testing. These effects may be due to the deficiency of stress fibers and pseudopodia in cinnamon-treated M1 macrophages (Figure 2A). Based on this observation of diminished pseudopodia, macrophage function (e.g., phagocytosis) was also tested.

The macrophages’ phagocytic capabilities under the influence of e-liquids were explored using *E. coli*, a Gram-negative facultative anaerobe serving as a proof-of-concept model to study the phagocytosis of bacterial pathogens, such as *P. gingivalis*. First, the amounts of macrophages measured by 18S qPCR amplification after phagocytosis assays remain consistent in terms of cells per well across groups. Therefore, the changes seen after host–bacteria interactions are not due to the loss of macrophages but rather due to e-liquid treatments. *E. coli*, measured by 16S qPCR amplification, allows for the calculation of the bacteria-to-macrophage ratio in order to evaluate the effects of e-liquids as compared to the control. The estimated total amounts of bacteria associated with macrophages (Table 1) indicate, as expected, that the majority of bacteria are extracellular. Quantification with qPCR accounts for both intracellular and extracellular bacteria bound to macrophages, as well as dead and live bacteria. However, CFU counting after exposure to killing media only accounts for intracellular live bacteria, which represents less than 3% of all of the estimated total *E. coli* (Table 1). This suggests that during *E. coli*–macrophage interactions, bacteria are both engulfed and attached to cell surface receptors on macrophages, where the latter remain extracellular and account for the vast majority throughout the experiment. E-liquid treatments severely decrease the rates of bacterial engulfment, most likely due to the lack of pseudopodia (Figure 4), which is also observed in Figure 2A. Interestingly, the flavorless e-liquid also significantly decreases, albeit to a lesser extent, the engulfment of *E. coli* (Table 1), suggesting that the PG/VG/nicotine mixture without flavors may also hinder phagocytosis. Similarly, others also show a decrease in *E. coli* and *Staphylococcus aureus* phagocytosis by human neutrophils [59,60] or *Haemophilus influenza* by THP-1-derived macrophages [22] when innate immune cells are exposed to electronic cigarette vapor delivered into the cell culture media.

Interestingly, cinnamon-treated macrophages are almost unable to engulf *E. coli* to the extent of M1 control, but are able to adhere to them at high rates (Table 1). TLR-4 expression may explain this observation since this receptor, which binds to Gram-negative bacteria, is upregulated following exposure to cinnamon e-liquid [51]. In other words, cinnamon e-liquid-treated macrophages bind to *E. coli* but fail to engulf it, presumably because these macrophages lack pseudopodia. This is supported by fluorescent microscopy (Figure 4) consistently showing cinnamon-treated M1 cells with the least amount of GFP+ *E. coli*. Previous investigations show similar results when looking at the effects of cinnamon-flavored e-liquids on macrophage phagocytosis. In a previous study, alveolar macrophages demonstrated a significant reduction in phagocytosis following exposure to multiple percent concentrations of “Sini-cide”, a specific cinnamon-flavored e-liquid, compared to the control [55].

*P. gingivalis* is a well-known pathobiont involved in the progression of periodontal disease. Some of its virulence mechanisms include production of gingipains as well as adherence and invasion of epithelial cells [61,62] and, most recently, escape from macrophage phagocytosis [38]. In addition, typically *P. gingivalis* does not kill the host cell upon escaping, as the bacteria use non-lytic exocytosis and recycling pathways, spreading from cell to cell without damaging the cell membrane [63,64,65]. Therefore, it was questioned as to whether or not escape from macrophages and subsequent invasion of OKF6/TERT-2 cells would be impacted when macrophages were exposed to e-liquid treatments. After exposing e-liquid-treated M1 macrophages to *P. gingivalis* testing phagocytosis and escape, the amount of intracellular *P. gingivalis* was lower, and the amount of escaping bacteria was greater when phagocytosed by M1 macrophages polarized with menthol and cinnamon e-liquids compared to M1 control (Figure 5). After escaping phagocytosis from cinnamon-treated macrophages, *P. gingivalis* invades oral epithelial cells to a greater extent compared to the control (Figure 5 and Table 2). This suggests that exposure to menthol- and cinnamon-flavored e-liquids results in compromised phagocytosis and retention, thus promoting *P. gingivalis* survival. Similar studies show that when exposed to nicotine, this oral pathogen shows a significant increase in invasion into epithelial cells [66]. Our previous studies showed that treatment with cinnamon-flavored e-liquid significantly reduced the expression of HLA DR and CD80 as well as the cytokines interleukin (IL)-1β, IL-6, IL-8 and TNFα, which hindered a proper immune response, as these cytokines and genes are crucial for the inflammatory process [51] and possibly phagocytosis. This could explain the exacerbated infections with *Pseudomonas aeruginosa* in mouse models exposed to electronic cigarettes reported in 2020 by Corriden and coworkers [60]. It is also worth pointing out that within periodontal disease, in addition to the antimicrobial role (e.g., phagocytosis), M1 macrophages also promote strong inflammation leading to tissue damage and bone resorption [67].

There are several limitations that should be considered when interpreting these findings. First, while THP-1-derived macrophages and OKF6/TERT-2 cells are innate immune and epithelial cell lines, respectively, this study did not include bone marrow-derived macrophages. Although OKF6/TERT-2 cells are an appropriate model to investigate *P. gingivalis*–epithelial cell interactions, primary gingival cells have been used to study such interactions [68,69,70]. Secondly, only *P. gingivalis* was investigated for escape and invasion, but these mechanisms could be investigated with other oral pathogens. It will also be informative to investigate *P. gingivalis*’s escape pathways. Third, this study does not include the separate effects of PG or VG or nicotine alone since these ingredients are typically present in e-liquids, but the individual testing of each could afford further insight into the PG/VG/nicotine combined effect. Fourth, the post-escape invasion process may present mechanisms that are similar to or different from the well-documented *P. gingivalis* invasion into oral epithelial cells [34,69]. Fifth, this investigation involves exposure of macrophages to e-liquids, but not bacteria. A recent study from our group indicates that *P. gingivalis* planktonic growth is severely inhibited by cinnamon- and menthol-flavored e-liquids [71], which could impact the outcomes of this investigation. This pathogen is able to invade epithelial cells through binding of fimbriae to β1 integrin receptor inducing the phosphorylation of paxillin [63]. Therefore exploring the interactions with this receptor could provide important information on the mechanism of invasion during e-liquid treatment. Finally, the *P. gingivalis* capsule also aids in avoiding the immune response [72], which could have also been explored under e-liquid treatment. Overall, this study addresses the effects of e-liquids on macrophage–bacteria interactions, specifically macrophage phagocytosis, escape and invasion. This study finds that after e-liquid exposure, macrophage morphology and phagocytosis are defective while *P. gingivalis* escape and invasion of OKF6/TERT-2 cells are increased. However, further investigation is required to address many of the above limitations.

In conclusion, this study demonstrates that under e-liquid exposure, M1 macrophage morphology and surface area are negatively impacted, with cinnamon-flavored e-liquid showing the greatest effect. This is characterized by reduced pseudopodia deviating from the typical M1 phenotype. This observation is concomitant with reduced macrophage function as the phagocytosis of both *E. coli* and *P. gingivalis* is diminished when exposed to flavored e-liquids. These host–bacteria interactions are even more impactful since *P. gingivalis* escapes from M1 macrophages and invades OKF6/TERT-2 cells. Further research is necessary to better understand these phenomena.

## Figures and Tables

**Table 1:** Macrophage–*E. coli* interactions with and without e-liquid treatments following phagocytosis assays.

	M1 Control	Flavorless	Cinnamon	Menthol
18S Ct values (macrophages)	25.72 ± 0.61	25.34 ± 0.69	25.69 ± 0.71	25.21 ± 0.71
18S 2^−ΔCt^	1.60 ± 0.43	2.27 ± 0.61	1.85 ± 0.54	2.27 ± 0.58
16S Ct values (*E. coli*)	23.87 ± 0.69	24.16 ± 0.72	24.90 ± 0.57	23.88 ± 0.71
−ΔCt (16S–18S)	−1.85 ± 0.15	−1.18 ± 0.10	−0.79 ± 0.28	−1.33 ± 0.21
Bacteria:macrophages (2^−ΔCt^)	3.74 ± 0.41	2.31 ± 0.15 *	1.95 ± 0.35 **	2.40 ± 0.38
Estimated total *E. coli* ^	748,900 ± 81,950	461,580 ± 30,435 *	390,505 ± 69,615 **	540, 540 ± 75,881
Intracellular *E. coli* ^&^	19,833 ± 1148	13,752 ± 1424 ***	77 ± 33 ***	5768 ± 827 ***
% Intracellular *E. coli* ^#^	2.65% ± 0.15	2.98% ± 0.31	0.02% ± 0.01 ***	1.07% ± 0.15 ***

^ Estimated total number of bacteria is calculated based on the ratio of bacteria:macrophages, where for macrophages there are 200,000 cells/well. ^&^ Intercellular *E. coli* quantified by CFU counting. ^#^ Percentage of intracellular *E. coli* = (intracellular *E. coli*/estimated total *E. coli*) × 100%. All values are presented as mean ± SEM (n = 8 to 13). Significance determined by One-Way ANOVA. * = *p* < 0.05, ** = *p* < 0.01, and *** = *p* < 0.001.

**Table 2:** Interactions between *P. gingivalis*, THP-1-derived M1 macrophages and OKF6/TERT-2 with and without e-liquid treatments.

	M1 Control	Flavorless	Cinnamon	Menthol
Intracellular *P. gingivalis* CFUs	274,394 ± 10,288	221,894 ± 20,277	115,438 ± 6063 ***	191,667 ± 14,919 **
Escaping *P. gingivalis* CFUs	75,680 ± 5226	96,480 ± 10,440	98,880 ± 8571	138,400 ± 7485 ***
% Escaping *P. gingivalis* CFUs ^#^	28 ± 1.90	44 ± 5.18	70 ± 4.99 ***	85 ± 7.25 ***
*P. gingivalis* CFUs invading into OKF6/TERT-2 cells	9183 ± 600	7825 ± 688	12,333 ± 1325 *	14,317 ± 1798

^#^ Percentage of escaping *P. gingivalis* = (escaping CFUs/intracellular CFUs) × 100%. All values are presented as mean ± SEM (n = 6 to 14). Significance determined by One-Way ANOVA. * = *p* < 0.05, ** = *p* < 0.01, and *** = *p* < 0.001.

## Data Availability

All data included in the manuscript.

## Supplementary Materials

The following supporting information can be downloaded at https://www.mdpi.com/article/10.3390/pathogens15080870/s1: Table S1: Interactions between *E.coli* and macrophages. Table S2: Interactions between Pg, macrophages and OKF6TERT-2.
